# Northwestward shifts in the locations of genesis reduce the lifetime of landfalling tropical cyclones in China

**DOI:** 10.1038/s41598-025-11996-7

**Published:** 2025-08-06

**Authors:** Shifei Tu, Ziyan Deng, Quanjia Zhong, Mei Liang, Jianjun Xu, Jingchao Long, Liguo Han

**Affiliations:** 1 Shenzhen Institute of Guangdong Ocean University, Shenzhen, China; 2 South China Sea Institute of Marine Meteorology, College of Ocean and Meteorology, Guangdong Ocean University, Zhanjiang, China; 3 Center for Ocean Research in Hong Kong and Macau (CORE), Department of Ocean Science, Hong Kong University of Science and Technology, Hong Kong, China

**Keywords:** Atmospheric science, Natural hazards

## Abstract

China is one of the countries most affected by tropical cyclones (TCs) worldwide. However, it remains unclear whether the significant poleward and coastal shifts in global TCs due to climate warming have affected the lifetime of landfalling TCs in China. Result shows that the lifetime of landfalling TCs in China has significantly decreased by 24% over the past 40 years, which is primarily contributed by the minor TCs. The duration in development stage has significantly shortened for both major and minor TCs. The northwestward migration of genesis locations has led to a decrease in duration of minor TCs, while the increase in the intensification rate has contributed to the shorter major TC durations. Further analysis suggests that these changes in TC lifetime and northwestward shifts are associated with a decrease in the Convective Available Potential Energy and westward extension of the subtropical high. The observed increase in rapid intensifications of major TCs is mainly driven by an increase in favourable thermodynamic environments. These findings clarify the connection between large-scale changes in TC genesis locations and regional characteristics of TC activity.

**Supplementary Information:**

The online version contains supplementary material available at 10.1038/s41598-025-11996-7.

## Introduction

Landfalling tropical cyclones (TCs) often cause severe disasters in coastal regions and countries^1–5^. Coastal regions of China are among the most severely affected regions worldwide, frequently impacted by TCs originating from both the western North Pacific (WNP) and the South China Sea (SCS)^6,7^. These TCs cause billions of dollars in direct economic losses each year^8,9^while the indirect economic losses caused by TC activities are incalculable^10,11^. In particular, several intense TCs, including Super Typhoon Doksuri (2305), Saola (2309), and Haikui (2311), hit most coastal cities in China. These TCs caused significant adverse impacts on agriculture, transportation, and infrastructure, as well as unquantifiable economic losses to metropolitan areas (such as Shenzhen, Hong Kong, Guangzhou, Shanghai, and their surroundings), mainly due to record-breaking heavy rainfall^12–15^. Therefore, it is crucial to have an in-depth understanding of their activities for disaster mitigation purposes^1,16^.

In general, about 7–8 TCs make landfall in China each year, and these landfall locations are mainly concentrated in southern and eastern China, with fewer occurrences in the northern regions of China, as the locations of genesis remain unchanged^1,17^. In fact, the locations of the lifetime maximum intensity (LMI) of global TCs are shifting significantly poleward and coastward under global warming^18,19^and these changes have profound implications for the pattern of TC activity in China^7^.

Since the 1980 s, there has been a significant increase in the destructiveness and damage caused by landfalling TCs over China, especially in eastern China, has been observed^16,20^which is associated with the increased frequency and intensity of landfalling TCs in this region, as well as the northward shifts in their tracks^21,22^. The compound hazard of landfalling TCs in eastern China has increased significantly, with the number of intense TCs doubling over the past two decades^23^. Correspondingly, the number of TCs affecting southern China has decreased, accompanied by a significant decrease in TC intensity over the past four decades^23,24^.

Recent studies have revealed substantial shifts in the spatiotemporal characteristics of TCs in the WNP. Significant poleward migration in the location of LMI has been attributed to changes in both TC genesis latitude and the distance between genesis and peak intensity^25^with weak TCs playing a dominant role in this shift^26^. The frequency of short-lived TCs has also increased over the past decades, contributing to a decrease in the average TC duration on a basin scale^27^. In particular, the lifetime of major TCs (Category 3 and above: LMI ≥ 96 kt) has shown a noticeable shortening trend, primarily associated with accelerated intensification and weakening rates during their life cycle^28^. Meanwhile, an increasing trend in the intensity of major TCs has emerged as a striking feature of global warming’s impact on TCs, whereas the intensity of minor TCs in the WNP exhibits a decreasing trend^29–31^. These findings suggest that large-scale environmental and thermodynamic changes are reshaping TC development and decay processes across the basin, including trends in duration and intensity.

Building on these basin-scale findings, it is important to understand how such shifts in TC behavior (such as duration) are reflected in landfalling TCs in China. However, the response of the duration of landfalling TCs in China to poleward and coastward shifts in global TCs remains unclear. In fact, TC duration not only affects disaster preparedness and mitigation efforts but also has significant implications for global and regional atmospheric water circulation. Therefore, we use historical best-track TC data from 1982 to 2021 to investigate changes in the duration of landfalling TCs in China.

## Results

### Observed short lifetimes of landfalling TCs in China

Since the beginning of the global satellite observation era in the 1980 s, the longest recorded duration of a landfalling TC in China was observed in 1986, when Typhoon Wayne (8614) remained at or above 35 kt for more than 18 days, according to the China Meteorological Administration (CMA) dataset. In contrast, Typhoon Koguma (2104) in 2021 had the shortest duration, reaching a tropical storm status near the Chinese coast before weakening rapidly within a few hours. In general, most landfalling TCs in China have durations of less than 10 days, with an average of approximately 111 h (~ 4.6 days) from 1982 to 2021, which is similar to the average duration of TCs in the WNP^32^.

**Fig. 1:**
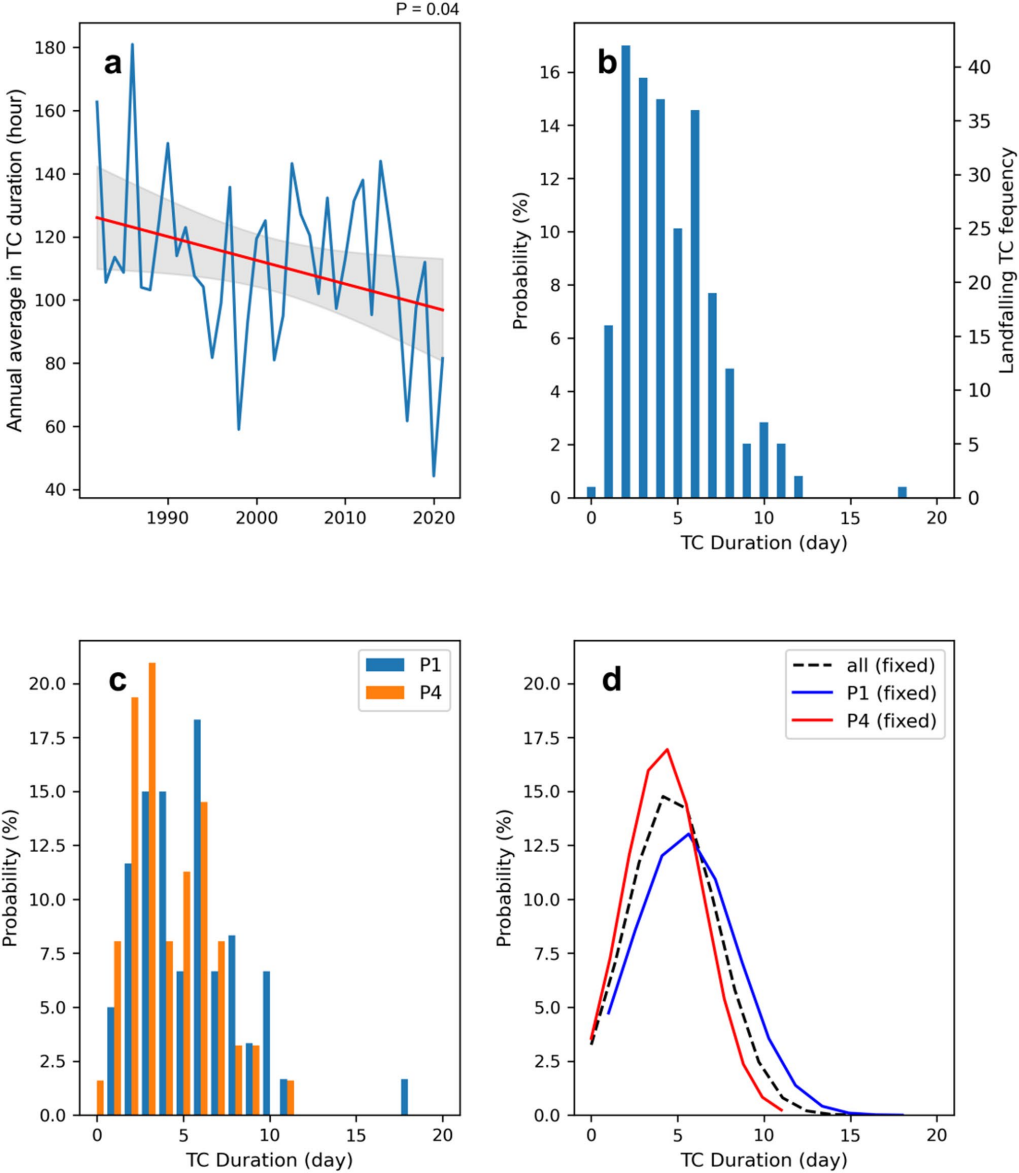
Decrease in the duration of landfalling TCs in China. (**a**) Time series of the annual average TC duration. The red line represents the linear trend, and the grey area indicates the 95% confidence interval. (**b**) Probability distribution function (PDF) and associated frequency of different durations of landfalling TCs. (**c**) Comparison of the PDFs of landfalling TC durations during the first and last decades (P1: 1982–1991; P2: 2012–2021). (**d**) Normal distribution fitting of the PDF of landfalling TC durations for the entire study period (black dashed line), P1 (blue) and P4 (red). All the results shown in these subfigures are obtained from the CMA dataset.

The annual average duration of landfalling TCs in China peaked at 180 h (7.5 days) in 1986 and then decreased sharply to 44 h (less than 2 days) by 2020. As shown in Fig. 1a, the duration shows a significant decreasing trend at a rate of about 7.5 h per decade. In the early 1980 s, the average duration was around 126 h, while it falls to less than 96 h in the 2020 s, with a 24% reduction. This pronounced decrease raises concerns about the potential effects of climate change, particularly in terms of regional impacts.

A similar decreasing trend in the annual average duration of landfalling TCs is also observed in other three additional datasets (Figure S1) from the Joint Typhoon Warning Center (JTWC), the Hong Kong Observatory (HKO), and the Japan Meteorological Agency (JMA), with slopes of −8.9, −5.2, and − 6.0 h per decade, respectively. Over the past 40 years, the average duration of TCs in the JTWC dataset has decreased by 26%, and those in the HKO and JMA datasets have decreased by 17% and 19%, respectively. The consistency across these different datasets further strengthens the robustness of the observed decreasing trend in the duration of landfalling TCs over China.

Further, we analyse the probability distribution function (PDF) of TC durations to better understand this trend (Fig. 1b). More than 85% of TC durations are within a week (excluding the tropical depression phase), while less than 10% extend to greater than 10 days. We find that TCs in the first decade (P1: 1982–1991) had longer durations, with 6 days being the most common (~ 18%), followed by 3–4 days (~ 15%). In contrast, durations of 2–3 days dominated (~ 40%) in the last decade (P4: 2012–2021), with a decreasing probability of duration of 4, 6, 8, or 10 days compared with P1 (Fig. 1c). The fitted normal distribution curves show a peak duration of 5–6 days in P1, which decreased to about 4 days in P4, strongly highlighting the shortening of TC life cycles over the study period (Fig. 1d). The average duration between these two periods shows a significant difference (*P* = 0.01).

### Duration in different classifications

**Fig. 2:**
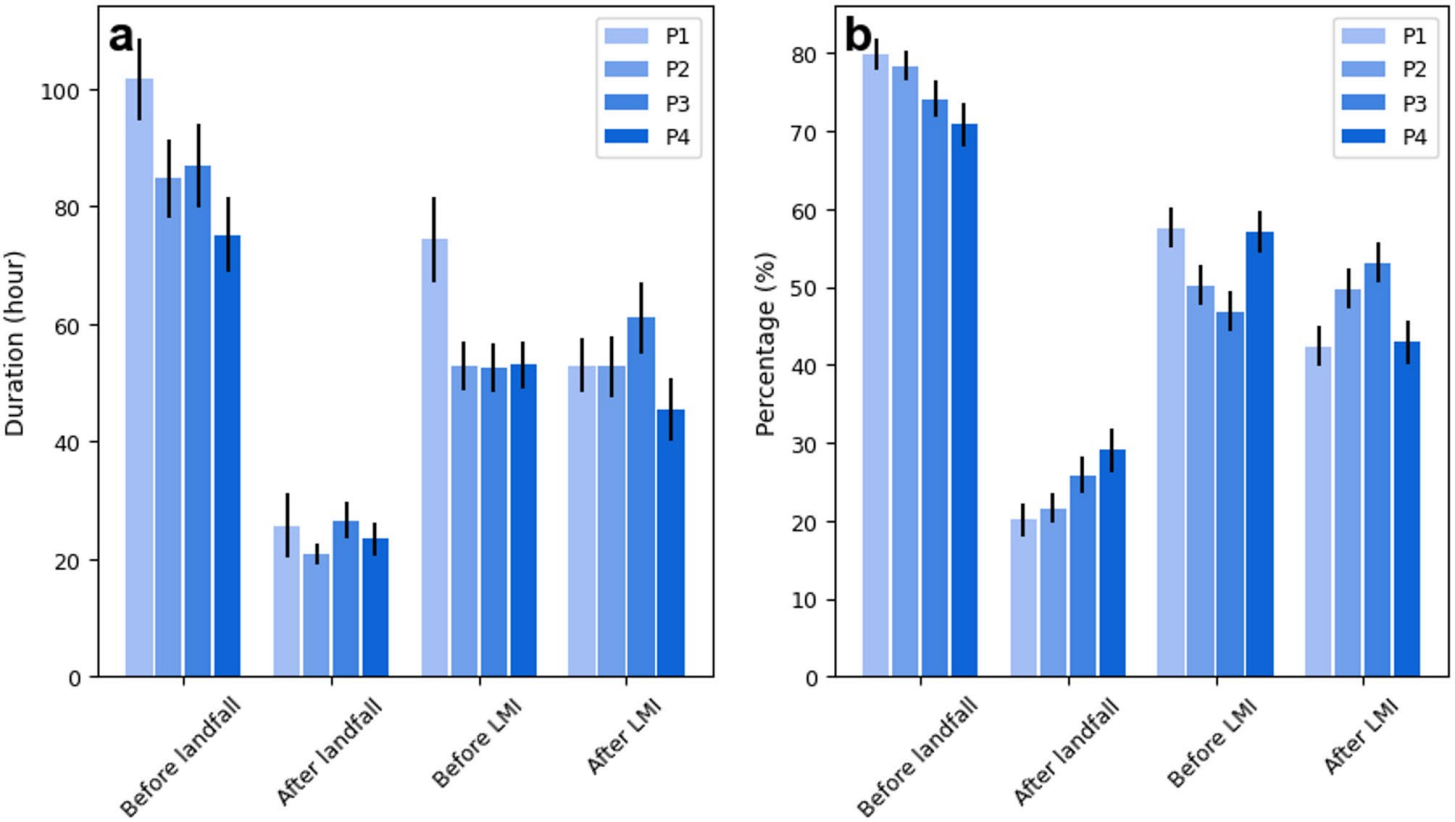
Comparison of the durations of different TC stages over the past four decades. (**a**) Duration of TCs in different stages (e.g., before and after landfall, before and after the LMI) over the four decades. (**b**) Percentage of the duration of different TC stages relative to the total lifespan. Vertical lines represent the standard errors.

**Fig. 3:**
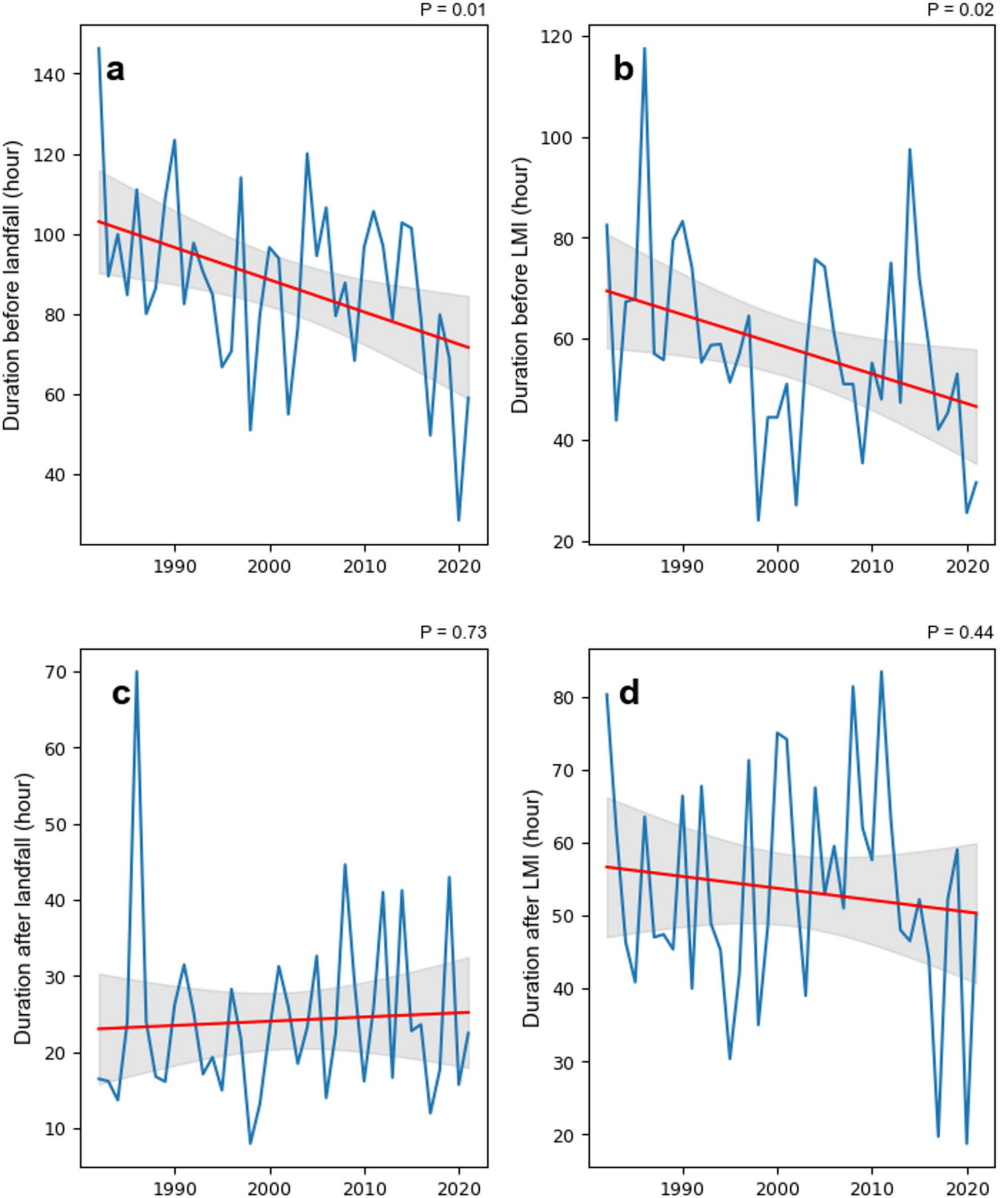
Changes in the duration of TCs making landfall in China during different TC periods. (**a**) Duration before landfall, (**b**) duration before the lifetime maximum intensity (LMI), (**c**) duration after landfall, (**d**) duration after the LMI. The red line represents the linear trend, and the grey area indicates the 95% confidence interval.

To better understand the changes in the durations of landfalling TCs in China over the past 40 years, we use two perspectives to classify TC stages: (1) before and after landfall, (2) before and after the TC first reaches the LMI (see Methods). From 1982 to 2021, the durations of TCs in different stages reveal significant interdecadal variations in the durations of TCs before and after landfall (Figs. 2 and 3). The duration before landfall in P3 (2002–2011) slightly increased compared with that in P2 (1992–2001); however, the trend remained downward throughout the entire study period (Fig. 3a). In contrast, the duration after landfall fluctuated without a clear trend (Figs. 2a and 3c). Notably, some TCs re-enter the sea surface after landfall under the influence of steering flow. For example, Typhoon Wayne in 1986 re-entered the sea after landfall, resulting in an exceptionally long duration. As a result, the average after landfall TC duration in 1986 reached about 70 h—around three times the other years. The decrease in duration before landfall shows a gradual decrease in the time from TC formation to landfall. This decrease is evident in the decreasing proportion of the duration of TCs before landfall, from 80% in P1 to 70% in P4 (Fig. 2b).

Changes in the TC duration before and after the LMI exhibited distinct patterns. In P1, the average duration before the LMI was about 75 h, which decreased to approximately 53 h in subsequent three decades, a decrease of 22 h (Fig. 2a). The decreasing trend in duration that occurred before the LMI over the last 40 years is notable (Fig. 3b), suggesting a significant shortening of the developmental stage of TCs. The corresponding proportion also decreased, although it increased in P4 because of the significant decrease in the LMI duration during this period (Figs. 2b and 3d). The after LMI TC durations in P1 and P2 were both around 53 h, while P3 exhibited the longest average duration at nearly 61 h. In contrast, P4 showed the shortest after LMI duration, averaging ~ 45 h. This overall pattern suggests a possible acceleration in the progression of TCs from genesis to peak intensity. However, the duration after the LMI stage does not exhibit a clear long-term trend, with only a slight decrease observed in the most recent decade. The shortened pre-LMI duration implies a faster intensification process, which may pose significant challenges for disaster preparedness related to landfalling TCs.

**Fig. 4:**
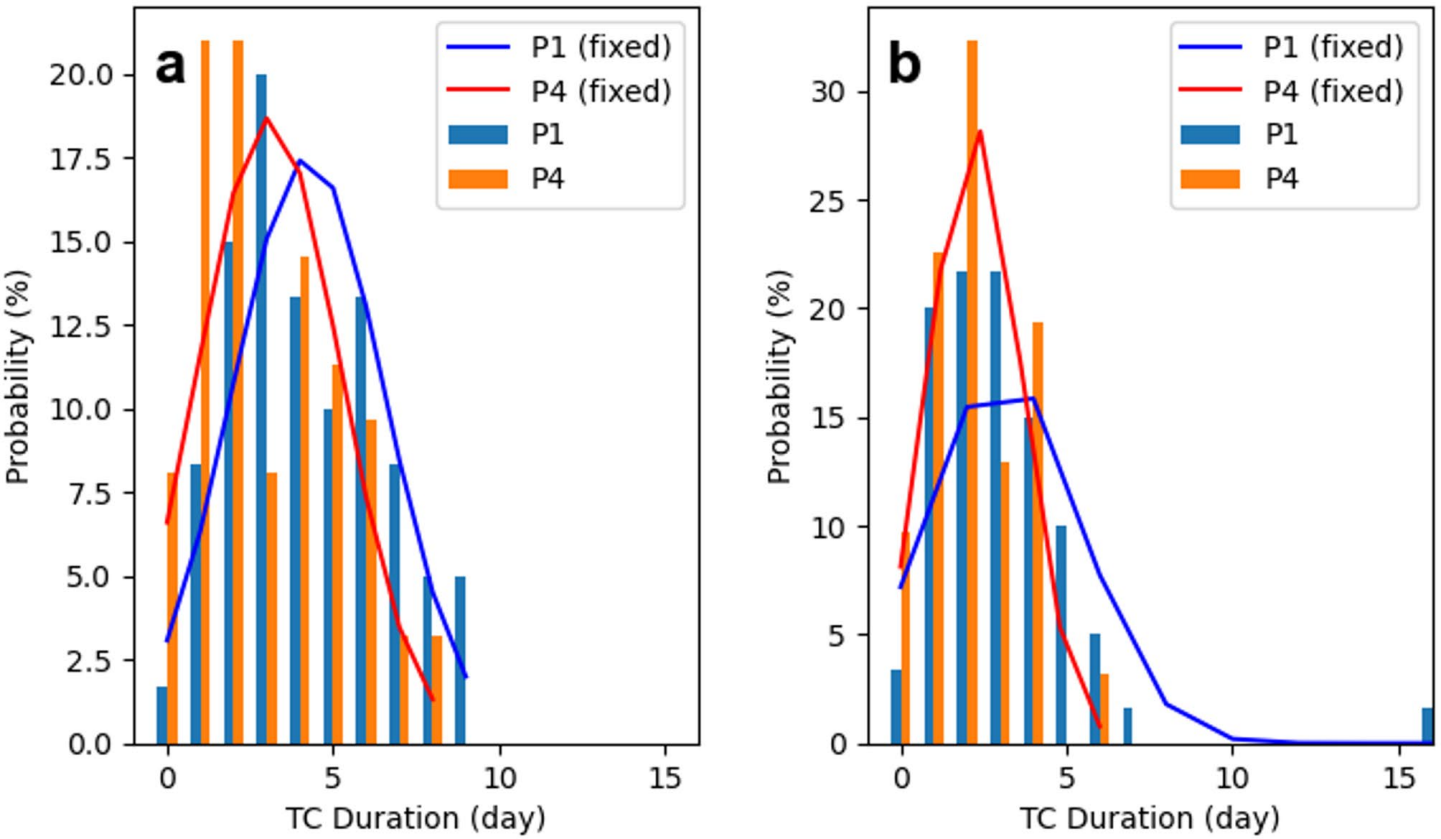
Comparison of PDFs of TC duration between the first and last decades (P1: 1982–1991 and P4: 2012–2021) with different TC classifications. (**a**) Duration before landfall, (**b**) duration before the LMI. The coloured curves represent the normal distribution fit of the sampled TC durations.

Further analysis of the PDF of the duration of landfalling TCs shows that the duration of landfalling TCs in China is predominantly within 10 days, with very few exceeding this threshold (Fig. 4). This pattern is particularly evident in the stages before landfall, and before the LMI is reached. The fitted normal curves of the PDF show that in P1, the average duration before landfall peaked at ~ 4 days but decreased to ~ 3 days in P4, reflecting a reduction of ~ 25%. The average duration between these two periods shows a significant difference (*P* = 0.01). This phenomenon means that more TCs complete their life cycle in a shorter time. Conversely, the duration after landfall remains stable and is predominantly concentrated within 2 to 3 days.

In the decay phase after the LMI of TCs, a slight shortening trend in duration is observed. In both periods, the distributions are concentrated primarily within 6 days. However, the proportion of the TC duration after the LMI at ~ 1 day increased significantly in P4, accounting for approximately 45% of the frequency. The significant decrease after the LMI duration in P4 may be attributed to the rapid transitions between the LMI and landfall (Fig. 3d). Previous studies have suggested that the duration between TCs reach their LMI and make landfall in China decrease rapidly, while the locations of the LMI shift closer to land^6^especially in the SCS^24^. These results are consistent with the observed global trends of poleward and shoreward migration of TC locations under climate warming.

**Fig. 5:**
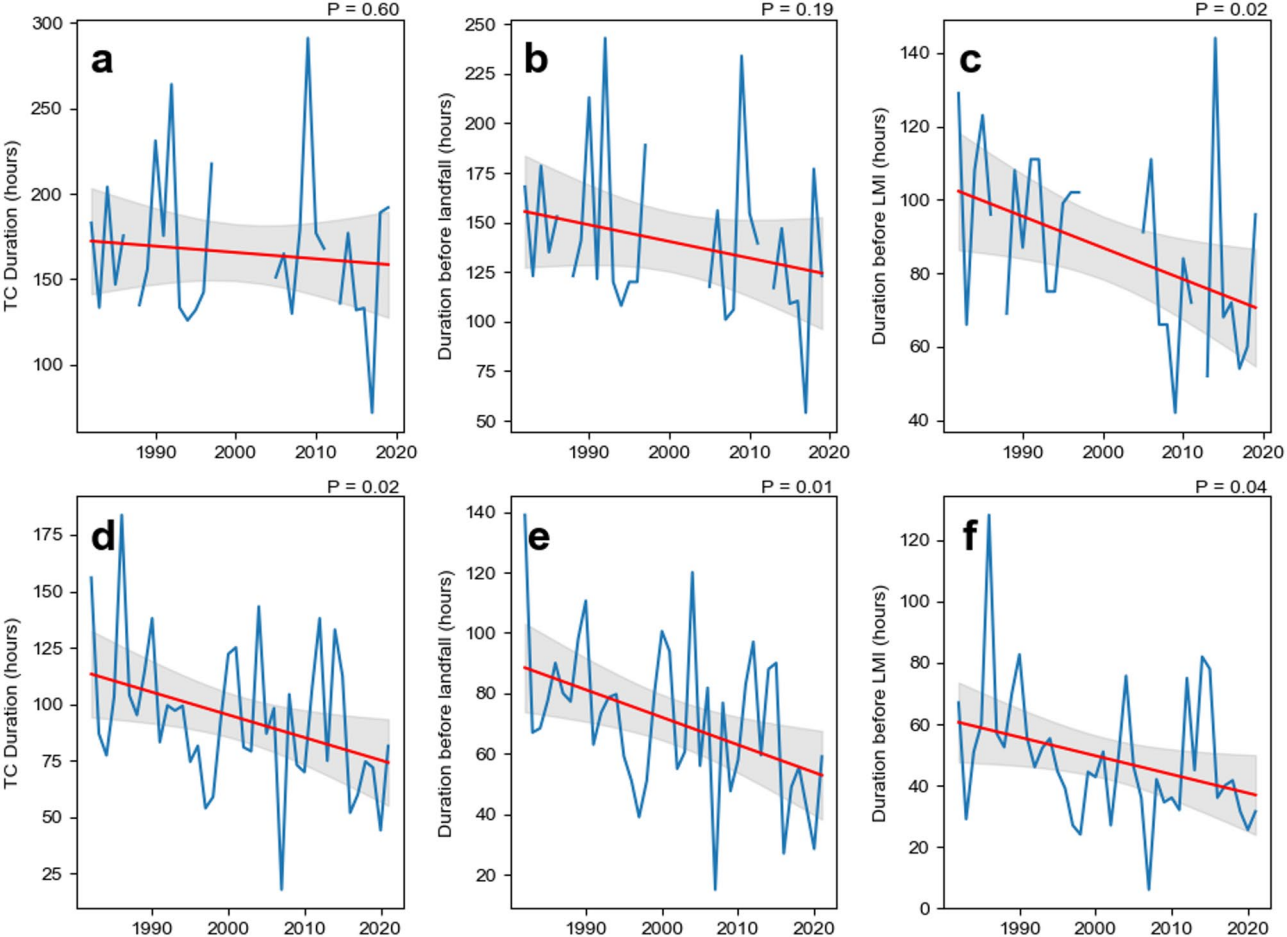
Changes in the duration of TCs making landfall in China during different TC periods. (**a**) Duration of major TCs, (**b**) duration before landfall of major TCs, (**c**) duration before the lifetime maximum intensity (LMI) of major TCs, (**d–f**) same as (**a–c**) but for minor TCs. The red line represents the linear trend, and the grey area indicates the 95% confidence interval.

Given that the response of TCs to climate change in the WNP region is highly dependent on their intensity, this study further examines the changes in the duration of major (LMI ≥ 96 kt) and minor (LMI < 96 kt) TCs based on their LMI classification (Fig. 5). The duration of major TCs making landfall in China shows a slight decreasing trend. Notably, the duration before landfall of major TCs has shortened more markedly (*P* = 0.19), while the duration before LMI has significantly decreased (*P* = 0.02). In contrast, minor TCs exhibit a statistically significant shortening in their total lifetime (*P* = 0.02), as well as in both the duration before landfall (*P* = 0.01) and before LMI (*P* = 0.04) stages.

These results further suggest that the reduction in the duration of landfalling TCs under climate change is more pronounced for minor TCs, and that even major TCs are experiencing shorter periods of intensification prior to landfall. This may reflect faster intensification and landfall processes in a warming climate, with important implications for early warning and disaster preparedness.

### Doubled occurrence of rapid intensification cases

Under the more favourable environmental conditions, such as increased ocean heat content and weakened vertical wind shear, a previous study has reported an increase in the number of rapidly intensification (RI) TCs near the coast of China^33^. However, trends in RI cases of landfalling TCs remain unclear. While the duration of landfalling TCs has decreased, the number of TCs and their average intensity and LMI show no significant trend during the past four decades (Figure S2). The shortening of the development time of TCs may partly explain the observed increase in RI events near coastal regions, as shorter lifespans may coincide with environmental conditions more conducive to rapid intensification.

**Fig. 6:**
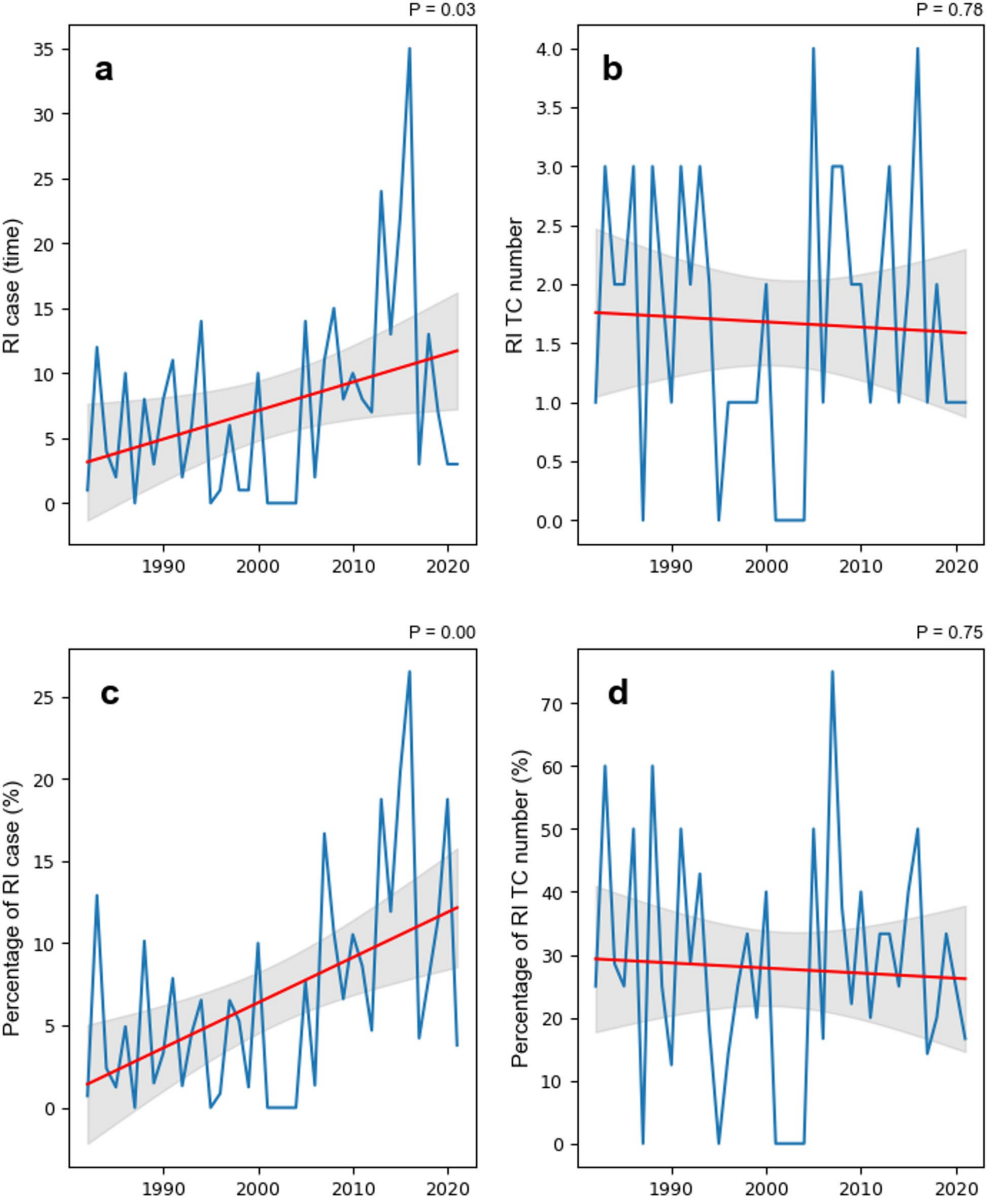
Changes in cases of rapid intensification (RI, 24 h intensity change > 30 kt) and RI TC number. (**a**) Number of RI cases, (**b**) Number of RI TCs, (**c**) Percentage of RI cases (RI cases/total cases), and (**d**) Percentage of RI TC number (RI TC number/total TC number). The red line represents the linear trend, and the grey area indicates the 95% confidence interval.

Statistical analysis revealed a significant increase in the total number of landfalling RI TC cases, defined as 24 h TC intensity increase ≥ 30kt (see Methods), in China from 1982 to 2022 (Fig. 6). The number increased from 59 cases in P1 to 130 cases in P4, representing a 2.2-fold increase compared with P1, indicating an increase in the duration or frequency of RI episodes per TC. As shown in Figure S3, the distribution of 24-hour intensity differences showed a significant increase in the RI cases. Although the total number of RI TCs did not significantly increase, the number of RI cases in each individual RI TC significantly increased (Fig. 6a), potentially exacerbating the destructive impacts of landfalling TCs. Furthermore, the proportion of RI cases among all TCs demonstrated a strong upwards trend (*p* = 0.00, Fig. 6c), indicating an increasing duration of TCs undergoing RI despite the total number of RI TCs without any significant trend (Fig. 6b and d). In other words, while the trend of RI TC frequency remained stable (Fig. 6b), the significant increase in the duration and proportion of RI cases highlights the evolving dynamics of TC intensification under changing environmental conditions. These results highlight the increasing importance and potential impact of RI cases on TC activity.

Analysis of RI cases exceeding 45 kt (the intense RI case) revealed a significant upward trend in both the number of such intense RI cases and the number of TCs that experienced RI (Figure S4). The proportion of intense RI cases among all TCs also increased notably, indicating that an increasing number of TCs are undergoing intense RI processes. Compared with RI cases exceeding 30 kt (Fig. 6), the number and proportion of TCs experiencing RI greater than 45 kt within 24 h significantly increased, highlighting a recent trend towards more intense RI processes.

Previous studies have shown that major TCs generally undergo RI, while minor TCs and tropical storms are much less likely to do so^34,35^. Similarly, our results reveal that RI cases associated with major TCs have increased in recent decades, and RI TC number also shows a slight increase (Figure S5). This increase in RI case reflects a faster intensification process, which in turn contributes to the observed shortening of the duration before LMI of major TCs.

The increase in the RI of landfalling TCs is consistent with previous work. Under a warming climate, both the frequency of nearshore RI TCs and the duration of RI events have been shown to increase globally^36,37^. Similar changes are also observed in the coastal areas of China, where the location of the LMI is moving progressively closer to the coast^6,24^.

### Possible influential factors

To better understand the reasons for the decrease in the duration of landfalling TCs in China from 1982 to 2021, we analysed the changes in their genesis locations, average locations, and LMI locations (Figure S6). The results reveal significant poleward migration of landfalling TCs (*P* < 0.05), which is consistent with global trends^18,38–40^. A significant westward migration of TC genesis location was also observed (*P* = 0.05). In terms of the distance from the TC centre to land, we find that the genesis locations of landfalling TCs have become slightly closer to the coast (*P* = 0.22). There are no significant trends in the average location or LMI location compared to land, which may be attributed to the complex sea‒land distribution in the offshore areas of China. However, the distance between the LMI and landfall locations has decreased significantly^6^which is consistent with the global coastal migration of LMI locations^19^.

**Fig. 7:**
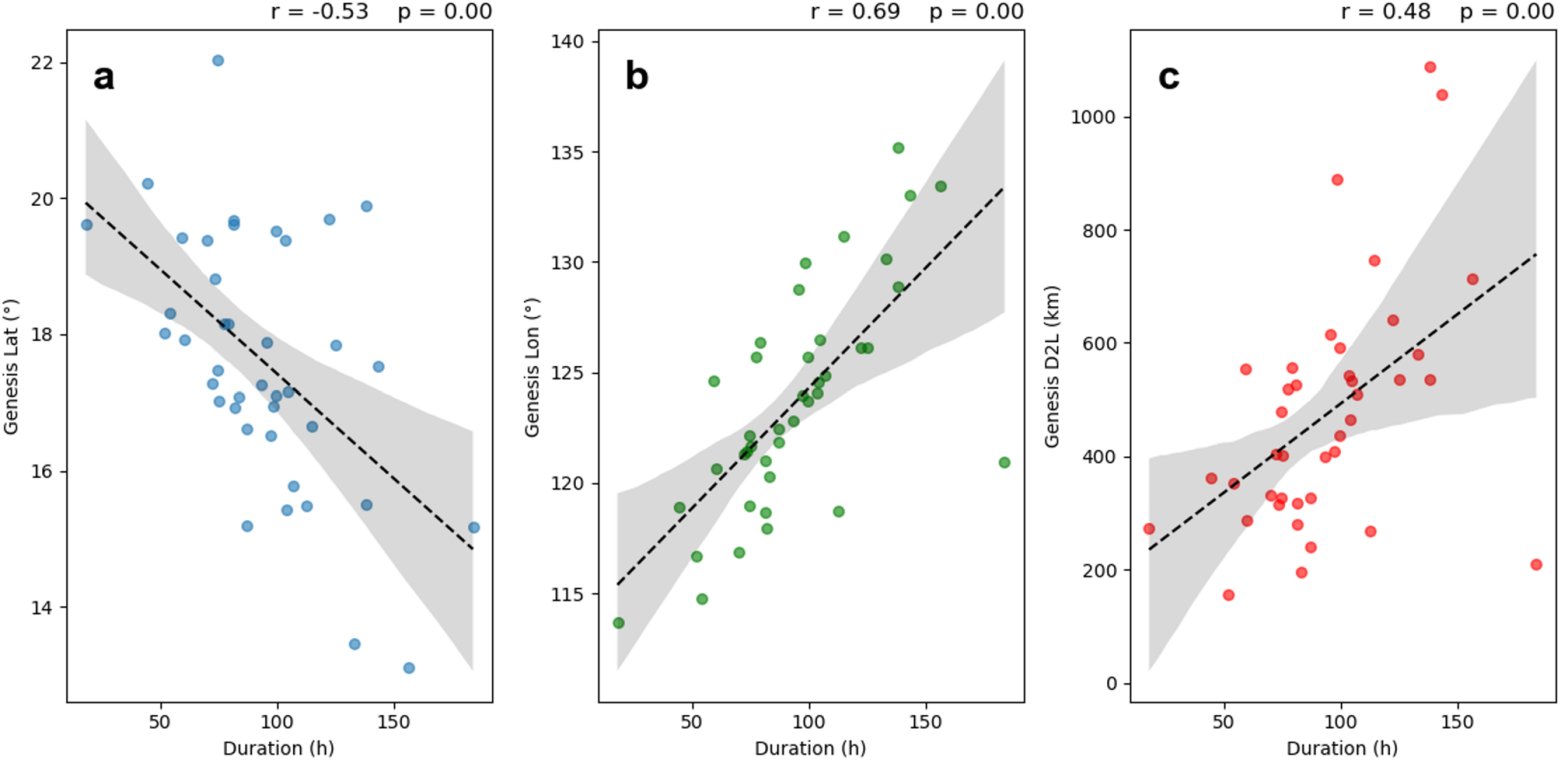
Correlations between the duration and genesis locations of minor TCs. (**a**) Duration vs. genesis latitude, (**b**) duration vs. genesis longitude, and (**c**) duration vs. the distance of genesis location to land. The black deashed line represents the linear trend, and the grey area indicates the 95% confidence interval.

Figure S7 shows the changes in genesis locations for both major and minor TCs. For major TCs, the poleward shifts in genesis latitudes are not statistically significant, which is consistent with findings across the broader WNP region^29^. However, the westward shifts in genesis longitudes are observed (*P* = 0.13), which may contribute to earlier landfalls of major TCs. In contrast, minor TCs exhibit significant northwestward shifts in genesis location (*P* = 0.02 and *P* = 0.04, respectively). Given the strong correlation between the duration and genesis location of minor TCs, shorter durations are associated with more northwestward in genesis locations and reduced offshore distance (Fig. 7). This shift appears to be a key driver of decreased TC duration.

**Fig. 8:**
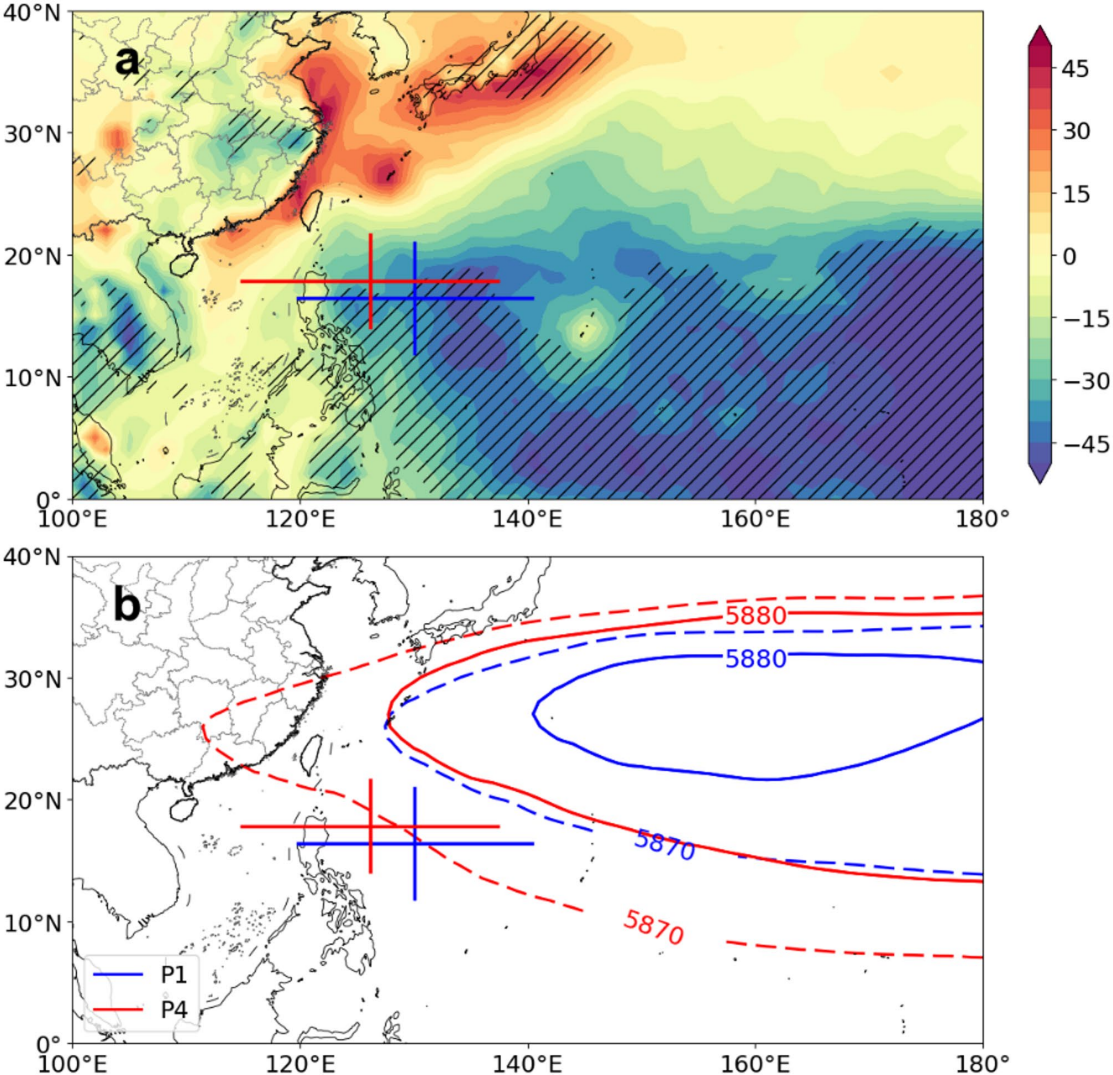
Composite differences in environmental conditions associated with changes in TC genesis locations between the first (P1) and last (P4) decades (P4 minus P1). (**a**) Convective Available Potential Energy (unit: J/kg) between the first (P1) and last (P4) decades. (**b**) Geopotential at 500 hPa during the first and last decades, with solid lines marking the 5880 isopotential and dashed lines marking the 5870 isopotential (unit: m). Blue and red represent the first and last decades. The administrative boundaries (national and provincial) were obtained from CN Open Data (https://www.cnopendata.com/) and visualized using Cartopy and Matplotlib in Python.

**Fig. 9:**
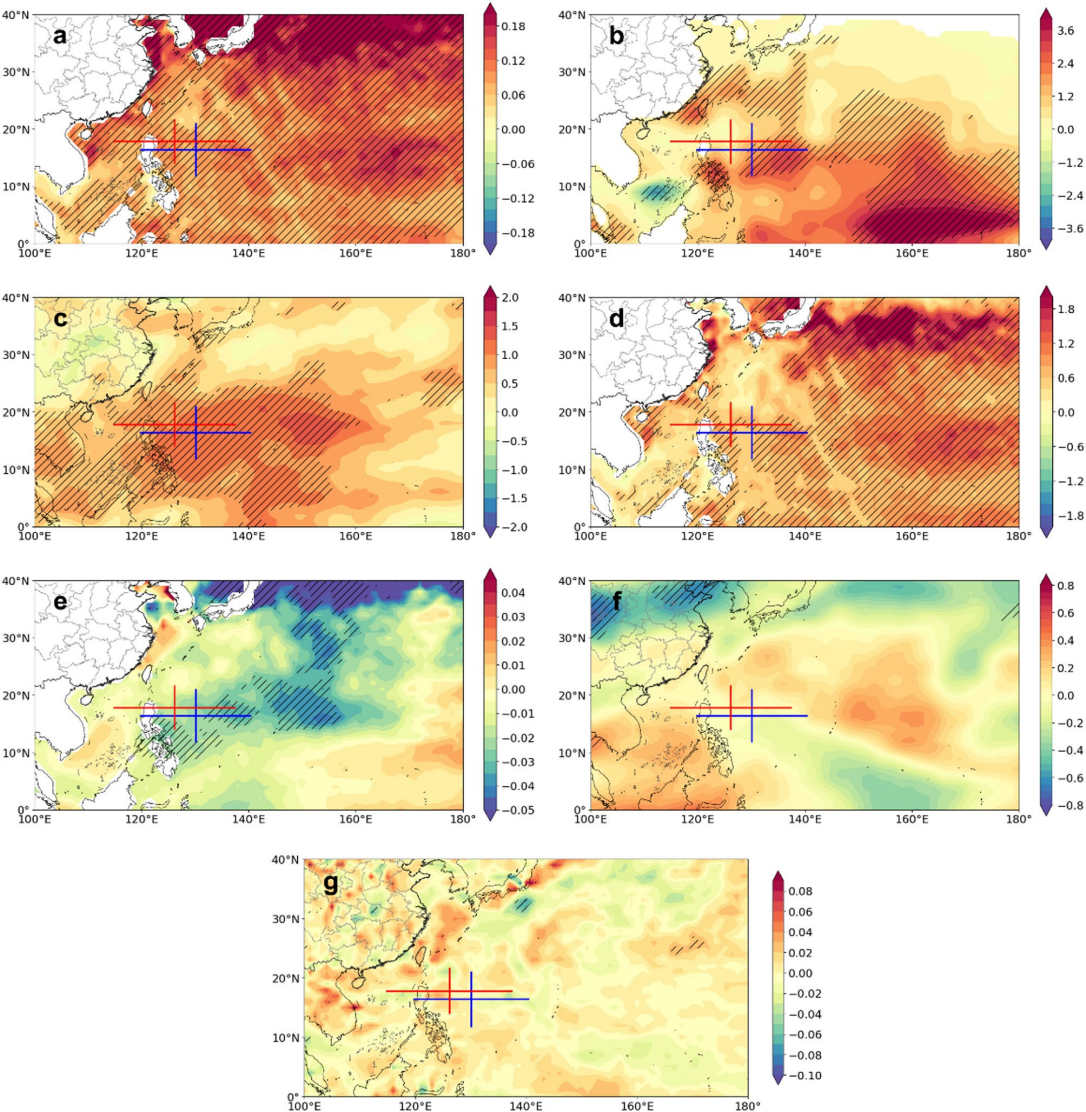
Composite differences in environmental conditions that influence TC RI, between the first (P1) and last (P4) decades (P4 minus P1). (**a**) Sea surface temperature (unit: °C), (**b**) TC heat potential (unit: J/m^2^), (**c**) relative humidity (unit: %), (d) Potential Intensity (unit: m/s), (**e**) moist entropy deficit (unit: J/kg K), (**f**) Vertical wind shear (unit: m/s), (**g**) relative vorticity (unit: × 10^4^ 1/s) at 850 hPa between the first (P1) and last (P4) decades. The shaded areas indicate significant differences at the 95% confidence level. The blue and red cross shape in all panels mark their average with their standard deviation of TC genesis locations during the P1 and P4 periods, respectively. The administrative boundaries (national and provincial) were obtained from CN Open Data (https://www.cnopendata.com/) and visualized using Cartopy and Matplotlib in Python.

Next, we further investigated how environmental factors influence the landfalling TC activity. In this study, we considered the possible influences of multiple thermodynamic and dynamic environmental conditions (Figs. 8 and 9). To avoid the influence of interdecadal variability, we focus on the differences in the environmental conditions between the first and last decades of the study period (P4 minus P1). Among these factors, only changes in Convective Available Potential Energy (CAPE) and the western North Pacific Subtropical High (WNPSH) show a significant association with the observed shifts in TC genesis. Specifically, the northwestward shift of TC genesis locations (especially for the minor TCs) is primarily attributed to the significant weakening of the CAPE over the eastern Philippine Islands (Fig. 8a), which suppresses TC formation in the region. Since the tracks of landfalling TCs in China are mainly influenced by the WNPSH, in this study, the variations in the intensity of the subtropical high during the P1 and P4 periods are further analysed in this study. As shown in Fig. 8b, the WNPSH intensifies and shifts westward from P1 to P4. This shift affects the location of TCs and has contributed to the increased proportion of tropical depression-stage TCs in recent years^24^.

Thermodynamic factors such as sea surface temperature (SST), TC heat potential, relative humidity, potential intensity (PI), and moist entropy deficit do not show a direct relationship with changes in the location of landfalling TCs in China (Fig. 9a–e). Instead, their influence is primarily reflected in changes in TC intensity. Although TC duration has decreased, our analysis shows no significant trend in either average intensity or LMI. However, the number of RI cases has increased significantly in recent decades. These changes in TC intensity and intensification rate are largely attributed to increasingly favourable environmental conditions—higher oceanic conditions, increased relative humidity, increased PI, and reduced moist entropy deficit—which are likely to have contributed to the observed increase in RI events. The increase in RI cases has, in turn, increased the challenges for forecasting, disaster prevention, and mitigation efforts for landfalling TCs in China. In contrast, vertical wind shear and 850 hPa relative vorticity show no significant changes during the study period, suggesting a limited influence on the observed variations in landfalling TCs (Fig. 9f–g).

### Implications for short lifetimes of landfalling TCs

**Fig. 10:**
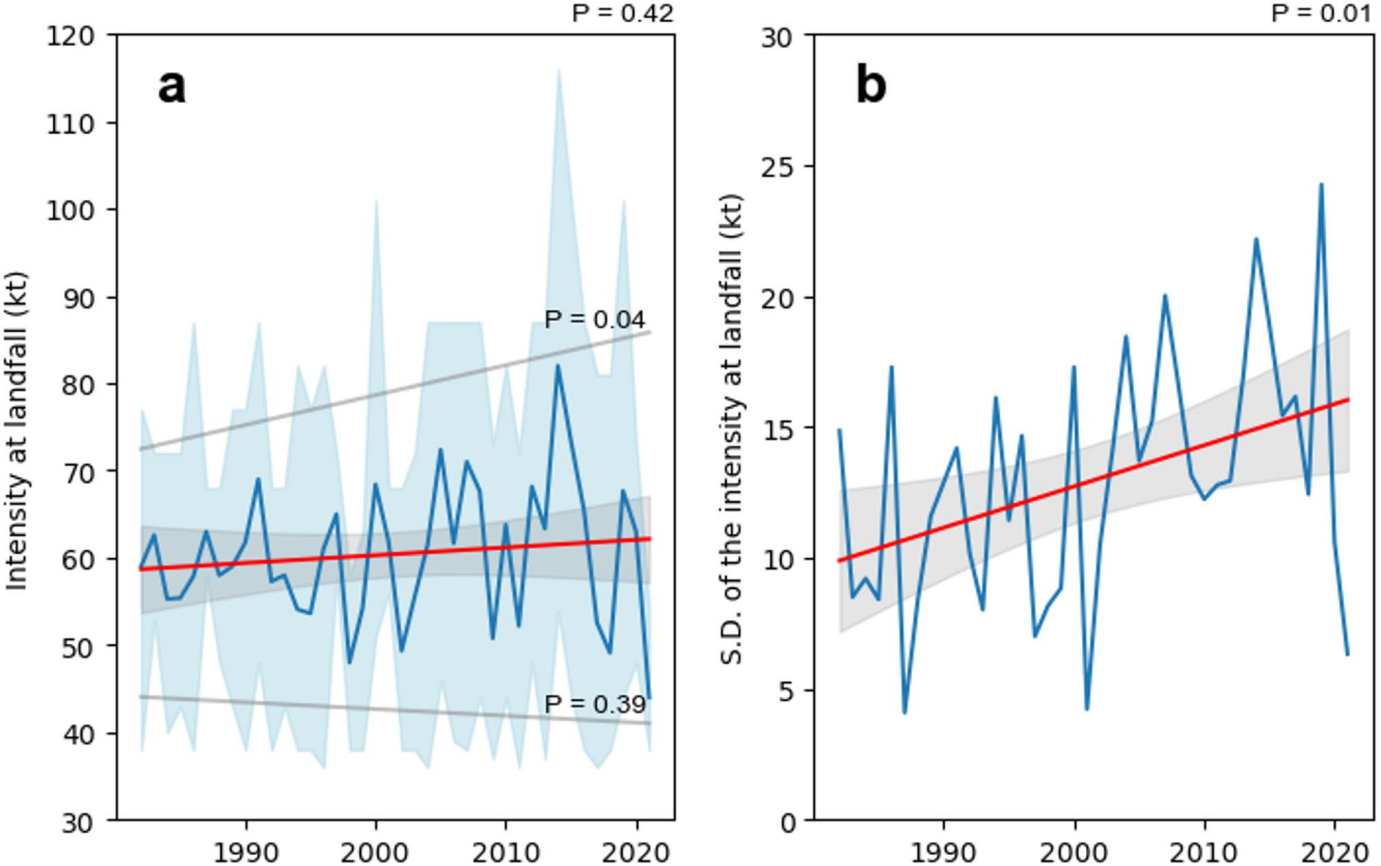
Changes in TC intensity at landfall over the last four decades. (**a**) Annual average landfall intensity of TCs, with the blue shaded area indicating the range between the maximum and minimum values of the landfall intensity, and the grey lines representing the linear trend of the maximum and minimum landfall intensities. (**b**) Annual standard deviation of TC landfall intensity in China.

In this study, a decreasing duration of landfalling TCs was observed, while their intensity without significant trend and the intensification rate increased. Figure 10a also shows a comparison of changes in TC landfall intensity over the last four decades. The minimum landfall intensity decreased slightly (*P* = 0.39), while the maximum landfall intensity increased significantly (*P* = 0.04). The average intensity at landfall also increased slightly (*P* = 0.42). The widening shaded area in Fig. 10a indicates an increasing amplitude of landfall intensity. Furthermore, the significant increase in the standard deviation of landfall intensity highlights the increasing uncertainty in TC intensity changes (Fig. 10b), which poses a greater challenge to disaster prevention for landfalling TCs. These changes are likely to be related to global climate change, including increasing SST and shifts in atmospheric conditions that influence TC development and landfall intensity^41–43^.

Meanwhile, the potential destructive changes in TCs are analysed. Despite the significant reduction in the annual average TC duration, the power dissipation index (PDI) and accumulated cyclone energy (ACE) of landfalling TCs remain unchanged (Figure S8a-b). This is primarily due to the maintenance of TC intensity under favourable SST and atmospheric water vapour conditions. A previous study revealed a significant increase in the PDI of landfalling TCs in mainland China, increasing the potential risks^20^. Our study also found a gradual increase in the average PDI and ACE for landfalling TCs over land (Figure S8c-d). This reflects a significant increase in the overland duration of TCs in mainland China^44^. At the same time, there is evidence of the northward shifts in landfall locations and affected areas, with more TCs now affecting the eastern, northern, and northeastern regions of China^21,45^.

Overall, the potential risk posed by landfalling TCs in China is increasing, driven by increased destructive potential and shifting impact areas. These trends highlight the importance of adaptation strategies to mitigate the growing threat of TCs under changing climate conditions.

## Conclusions

In this study, the changes in the duration of landfalling TCs in China from 1982 to 2021 are analysed, showing a significant decrease in their annual average duration, from about 126 h in the early 1980 s to less than 96 h in the 2020 s (with an ~ 24% reduction). Comparative analyses of TC duration classifications showed that the most significant shortening occurred before landfall, and before the LMI was reached. Regarding TC intensity classification, the overall decrease in TC duration is primarily contributed by minor TCs, which account for the majority of landfalling TCs in China. However, both major and minor TCs exhibit significant shortening in their developmental stages. To ensure that the recent increase in short-lived TCs^27^ dose not influence our findings, we conducted an additional analysis excluding TCs with durations less than two days. The results still show a significant shortening in TC duration before landfall and before LMI periods (Figure S9), confirming the robustness of our findings.

Attribution analysis suggests that the shortened duration of landfalling TCs in China is primarily driven by the northwestward shifts in genesis locations, which is closely associated with the weakened CAPE over the southeastern WNP and the strengthening and westward extension of the WNPSH. While the average TC intensity remains unchanged, the increased number of RI cases of major TCs is attributed to increasingly favourable thermodynamic conditions such as higher SST, increased relative humidity and increased potential intensity. These factors contribute to changes in TC duration, increasing uncertainty in landfall intensity and potential risk.

The significant northwestward shifts in locations of TC is accompanied by an increase in the number of TCs making landfall in eastern China^22,46^which increases the potential risk of TC impacts in the eastern, northern and northeastern regions of China. Our results improve our understanding of the mechanisms driving changes in TC landfalls under climate change.

In line with Wang et al.^28^we demonstrate that major TCs in China have also experienced a significant reduction in the duration of development stage, accompanied by comparable changes in intensification rates, and the increased number of RI cases is closely associated with this accelerated development stage. Furthermore, we highlight the critical role of poleward and coastward migration of TC locations in shortening in their lifetimes, especially for the minor TCs.

In fact, due to the poleward migration of TCs, similar phenomena of shortened duration may be observed for landfalling TCs in other regions, particularly for the minor TCs. Under the influence of climate change, the poleward migration of TCs significantly increases the potential risk to mid- to high-latitude regions^18,38,47^. Alongside with the increased risks associated with rapid changes in TC intensity^28,48,49^future research should focus on the effects of climate change on global and regional TC activity, particularly landfalling TCs.

## Methods

### Data

To investigate changes in landfalling tropical cyclones (TCs) in China, this study used the best track data from the International Best Track Archive for Climate Stewardship- China Meteorological Administration (IBTrACS-CMA), as this dataset has more reliable and detailed observations for offshore China^50,51^. Figure S10 shows the tracks of all TCs used in this study. The variables used included TC intensity (wind speed), location (latitude and longitude), and distance from TC centre to land. To verify the robustness of the results based on the CMA track data, records from the Joint Typhoon Warning Center (JTWC), Hong Kong Observatory (HKO), and Japan Meteorological Agency (JMA), as included in IBTrACS, were also used to analyse TC duration. The study period was 1982–2021, and to analyse the evolution of landfalling TCs in China, we calculated the TC characteristics at different decadal scales separately.

In addition, ERA5 reanalysis data^52^ from 1982 to 2021 were used to examine the potential impacts on landfalling TCs in China. These analyses include variables such as sea surface temperature (SST), relative humidity, relative vorticity, wind fields, water vapor, Convective Available Potential Energy (CAPE), and geopotential energy. The EN4.2.2 ocean analysis dataset^53^ obtained from Met Office Hadley Centre are used to investigate the oceanic conditions contributing to TC activity.

### Parameters of landfalling TCs in China

In this study, we selected landfalling TC samples based on a threshold intensity of ≥ 35 kt. The durations and TC-related parameters analysed were restricted to TCs recorded with an intensity of 35 kt or greater. Among the best track data provided by the four institutions, records with intensities less than 35 kt were excluded: all tropical depressions, as well as all TCs that weakened to less than 35 kt either before landfall or at the time of landfall, were excluded from the analysis. Therefore, the annual change in the TC frequency includes only landfalling TCs with an intensity of 35 kt or higher across China.

The duration of a TC is defined as the period during which the TC maintains an intensity of 35 kt or higher. During this period, key parameters such as TC intensity, lifetime maximum intensity (LMI), and their corresponding positional changes are recorded on an annual scale. The landfall intensity of a TC is defined as its intensity at the moment of first landfall in China.

### Different TC stages

To better characterise the temporal evolution of TC duration, we applied two complementary temporal classification frameworks: (1) the stages before and after the first landfall in China, and (2) the stages before and after TC first reaches the LMI. The former highlights the role of land interaction in modulating TC duration and is directly relevant for disaster preparedness and impact assessment. The latter captures structural and dynamical changes around the time of peak intensity, which are closely linked to storm evolution and potential destructiveness. Together, these two classifications provide a comprehensive understanding of the behaviour of TCs at different stages and offer new insights into long-term changes in their evolution.

### Rapid intensification of landfalling TCs

Following the conventional definition, a rapid intensification (RI) TC is defined as a TC intensity increase ≥ 30 kt within 24 h^54,55^. In this study, we quantified both the number of RI TCs and the number of RI cases (i.e., a case indicates a 24 h intensity change) among landfalling TCs in China each year. In addition, to investigate more intense cases of RI TCs, we analysed the number and cases of RI TCs associated with an intensity change ≥ 45 kt within 24 h.

### Environmental factors

To investigate the potential environmental drivers of TC activity, we analysed the changes in relevant environmental factors during the P1 and P4 periods. Specifically, SST, relative humidity at 700 hPa, relative vorticity at 850 hPa, geopotential height at 500 hPa, and CAPE were obtained directly from the ERA5 reanalysis dataset. Vertical wind shear was derived from the difference between wind vectors at 200 hPa and 850 hPa. In addition, potential intensity (PI) and moist entropy deficit were calculated following established methods from previous studies^56–58^. The calculation of TC heat potential was referring from previous studies^59,60^.

### Destructive potential of landfalling TCs

To describe the combined destructive characteristics of landfalling TCs in China, the power dissipation index (PDI) and accumulated cyclone energy (ACE) were used in this study. The PDI is defined as the time integral of the cube of the TC intensity (wind speed)^59–61^whereas the ACE is defined as the quadratic sum of the TC intensity^27,62,63^. In this study, we primarily calculated the average annual PDI and ACE for individual TCs, analysing both the total duration of the TC and the overland maintenance phase after landfall separately. (Note: All results were obtained for TCs with intensities ≥ 35 kt).

## Electronic supplementary material

Below is the link to the electronic supplementary material.

Supplementary Material 1

## Data Availability

TC best-track data are taken from the IBTrACS v4 dataset (https://www.ncdc.noaa.gov/ibtracs/). Contributing factors to TC rain rate are taken from ERA5 reanalysis (https://cds.climate.copernicus.eu/cdsapp#!/home). These datasets are publicly available.
