# Supplementary material for: Northwestward shifts in the locations of genesis reduce the lifetime of landfalling tropical cyclones in China
Source: Sci Rep. 2025 Aug 6;15:28764. doi: 10.1038/s41598-025-11996-7 (PMC12328659; doi:10.1038/s41598-025-11996-7)
Supplement: Supplementary file 1 — Supplementary Material 1 [file 41598_2025_11996_MOESM1_ESM.docx]

Supplementary Information

**Northwestward Shifts in the Location of Genesis Reduces the Lifetime of Landfalling Tropical Cyclones in China**

Shifei Tu^1,2^, Ziyan Deng^2^, Quanjia Zhong^3*^, Mei Liang^2*^, Jianjun Xu^1,2^, Jingchao Long^2^, Liguo Han^2^

1. Shenzhen Institute of Guangdong Ocean University, Shenzhen, China.

2. South China Sea Institute of Marine Meteorology, College of Ocean and Meteorology, Guangdong Ocean University; Zhanjiang, China.

3. Center for Ocean Research in Hong Kong and Macau (CORE) and Department of Ocean Science, Hong Kong University of Science and Technology; Hong Kong, China

(submitted to ***Scientific Reports***)

July 12, 2025

*Corresponding authors:

Quanjia Zhong, and Mei Liang, Email: [zqj@lasg.iap.ac.cn](mailto:zqj@lasg.iap.ac.cn); [liangmei@gdou.edu.cn](mailto:liangmei@gdou.edu.cn)


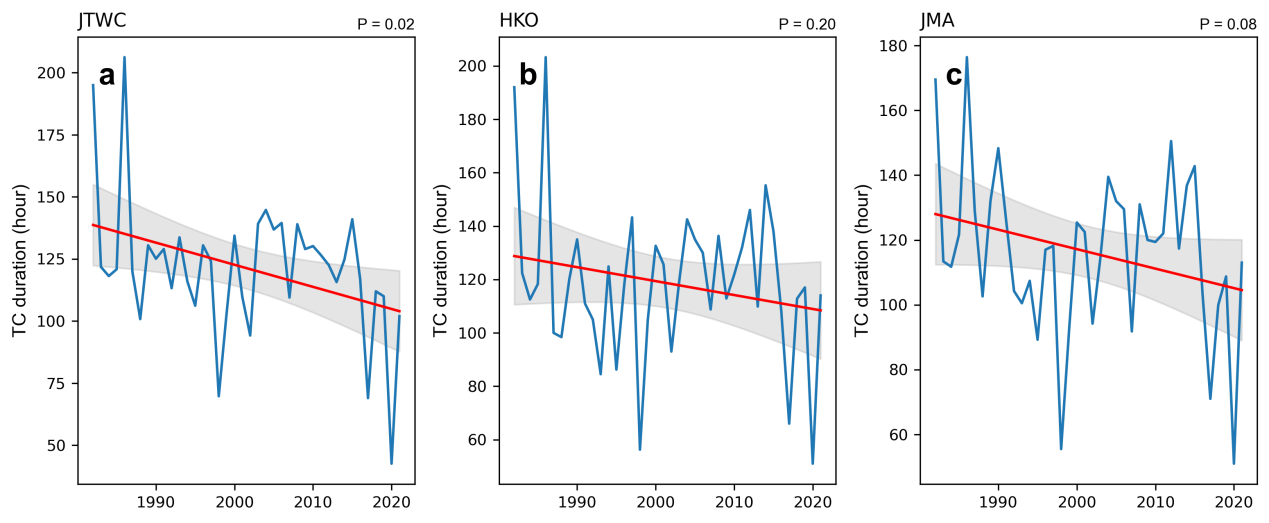


**Figure S1 | Changes in the durations of landfalling tropical cyclones (TCs) in China obtained from different TC datasets.** (a) JTWC, (b) HKO, and (c) JMA. The red line represents the linear trend, and the grey area indicates the 95% confidence interval.


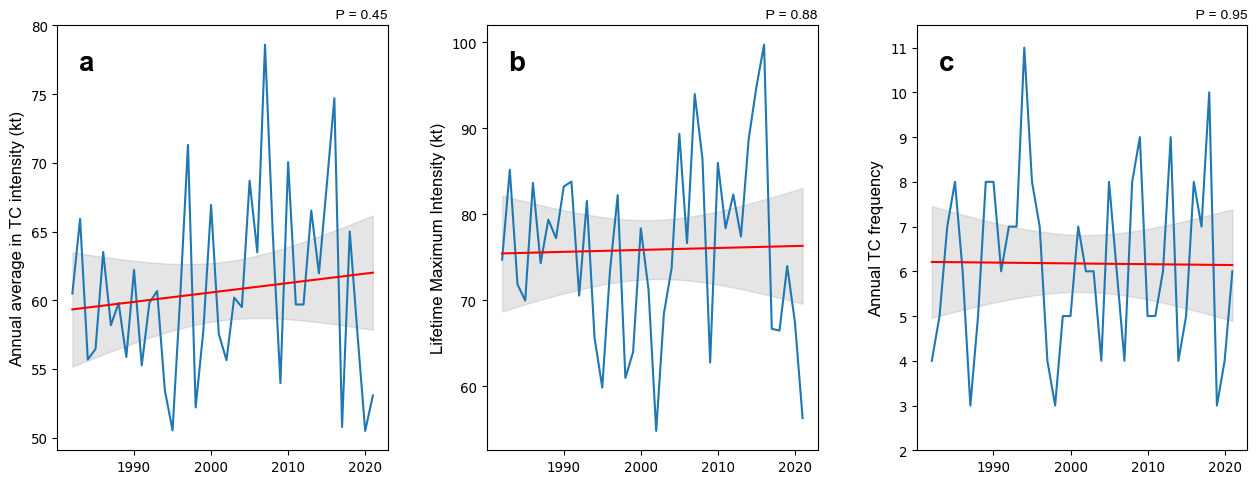


**Figure S2 | Changes in the TC intensity and lifetime maximum intensity.** (a) Annual average TC intensity and (b) lifetime maximum intensity (LMI). (c) Annual TC frequency. The red line represents the linear trend, and the grey area indicates the 95% confidence interval.


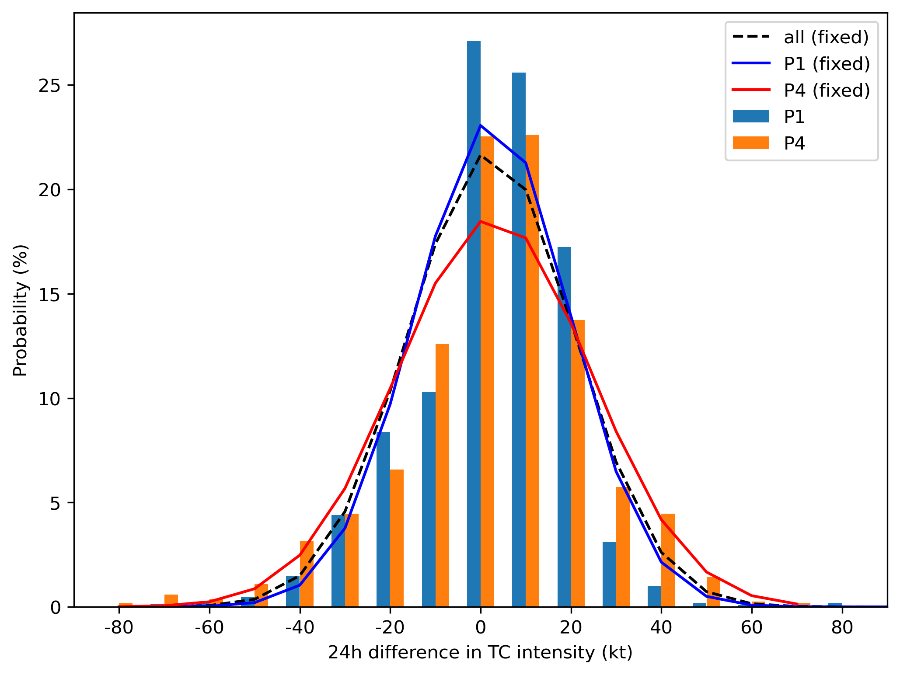


**Figure S3 | Comparison of the probability distribution functions of the 24 h intensity changes.** The blue and orange bars represent the first and last decades (P1 and P4), respectively. The black, blue and red curves represent the normal distribution fitting of the 24 h intensity change during the entire study period, P1 and P4, respectively.


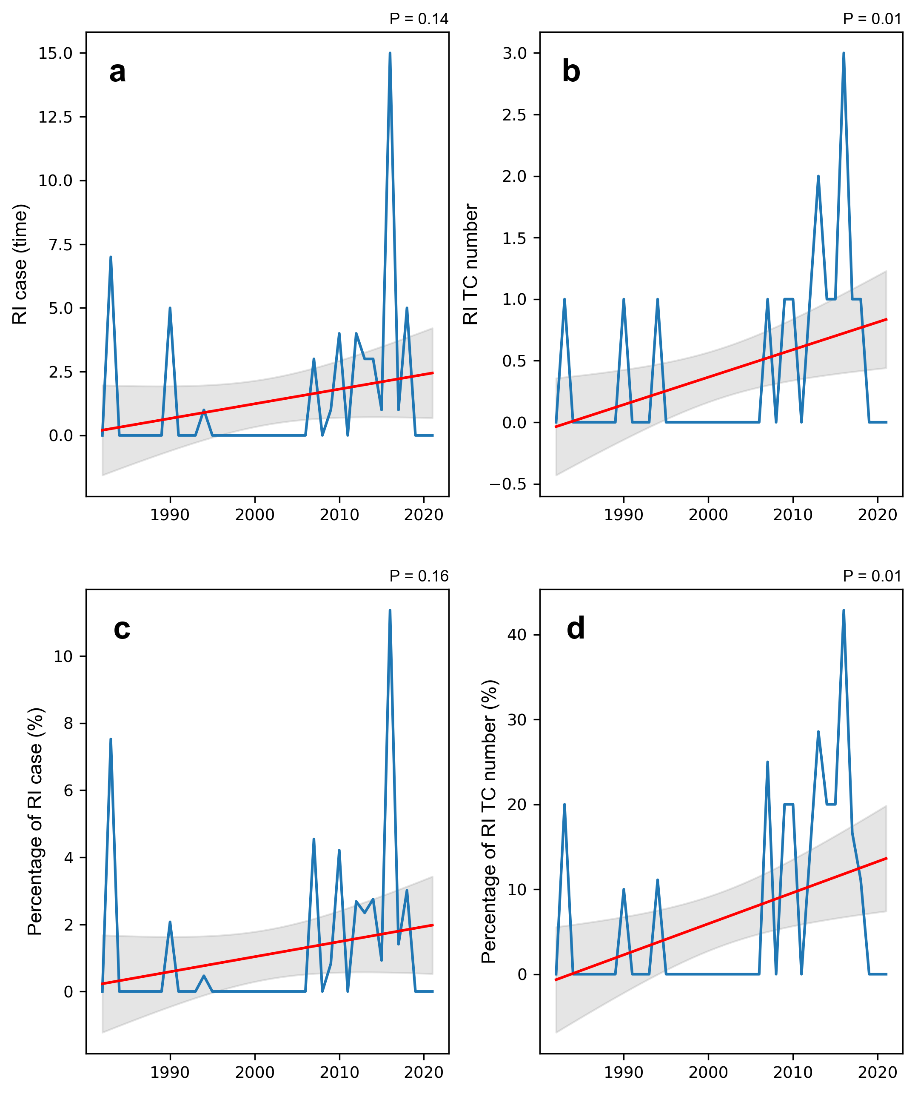


**Figure S4 | Changes in intense rapid intensification (intense RI, > 45 kt) cases and TC number.** (a) Number of RI cases (i.e., 24 h intensity change), (b) number of RI TCs, (c) percentage of RI cases (RI cases/total cases), (d) percentage of RI TC numbers (RI TC number/total TC number). The red line represents the linear trend, and the grey area indicates the 95% confidence interval.


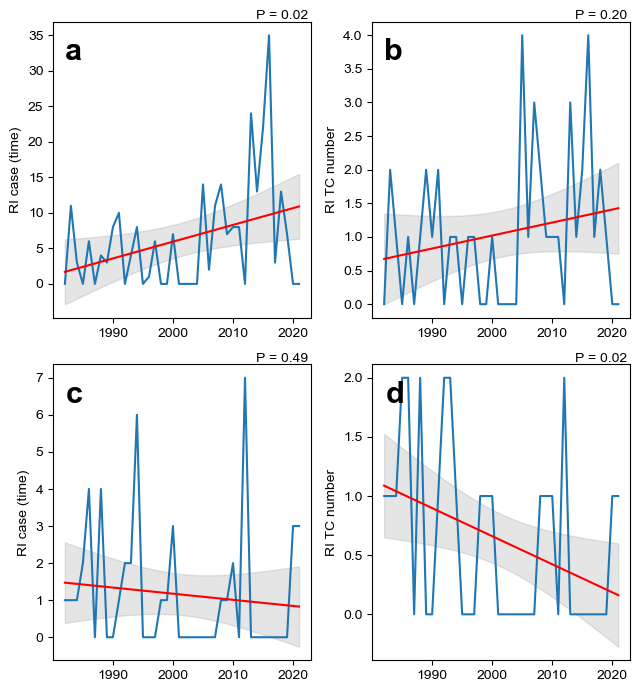


**Figure S5 | Changes in cases of rapid intensification (RI, 24** **h intensity change** ≥ **30 kt) and RI TC number for the major TCs.** (a) Number of RI cases, (b) Number of RI TCs. The red line represents the linear trend, and the grey area indicates the 95% confidence interval.


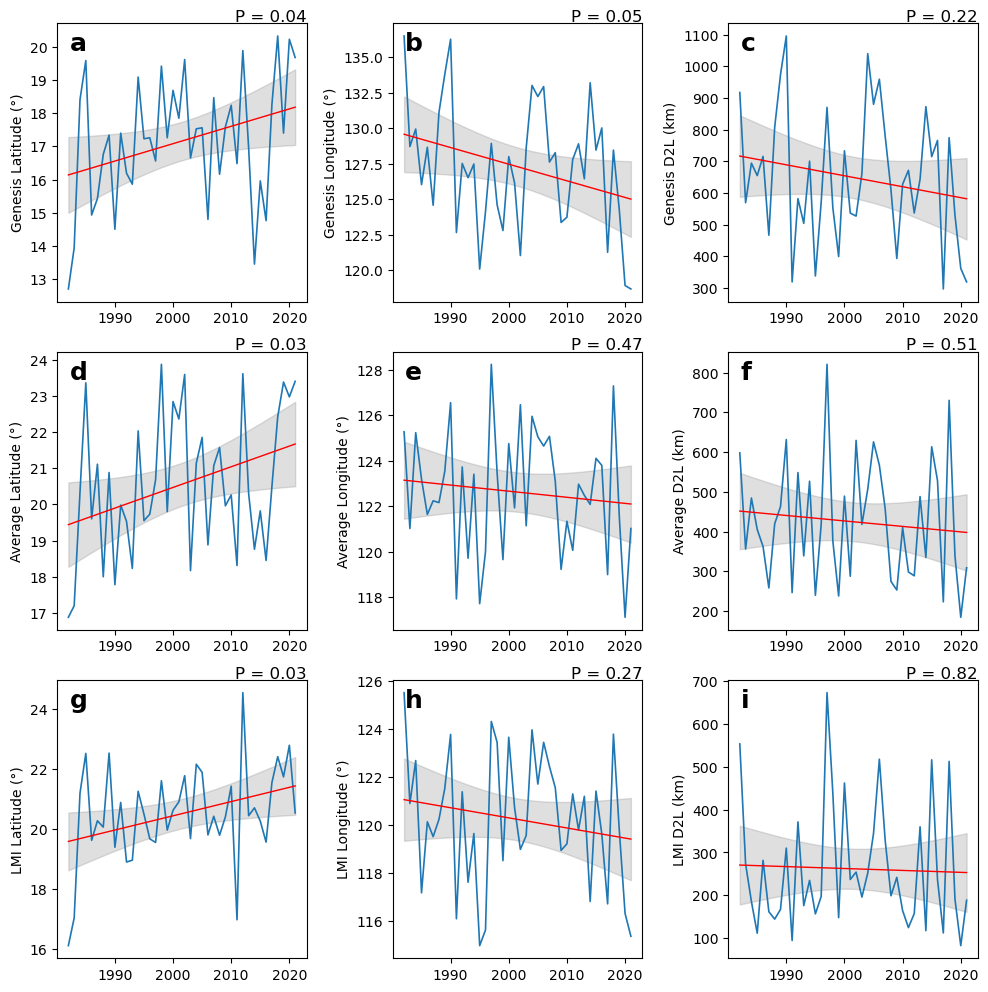


**Figure S6 | Comparison of the locations of landfalling TCs.** Annual averages of (a) latitude, (b) longitude and (c) distance to land for locations of TC genesis. (d-f) are the same as (a-c), respectively, but for the average locations of TCs. (g-i) are also the same as (a-c), but for the first location, TCs reach their lifetime maximum intensity (LMI). The red line represents the linear trend, and the grey area indicates the 95% confidence interval.


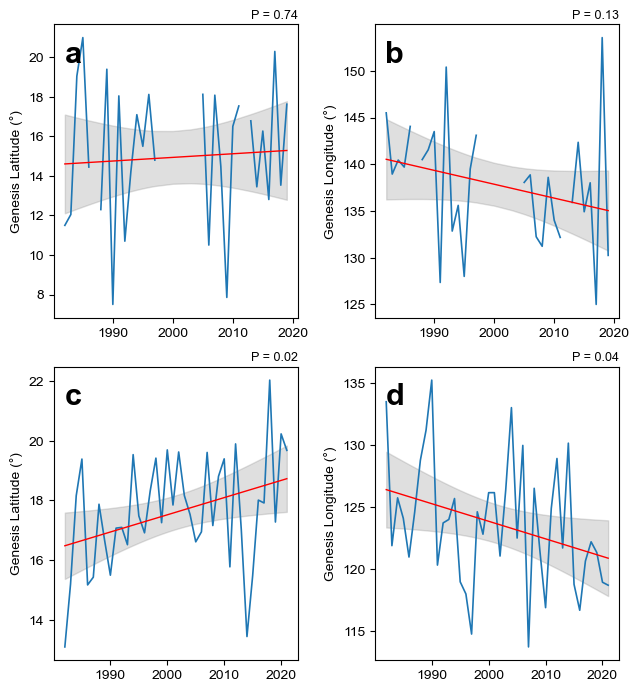


**Figure S7 | Comparison of the locations of landfalling major and minor TCs.** Annual averages of (a) latitude, (b) longitude of major TCs, (c-d) same as (a-b) but for the minor TCs. The red line represents the linear trend, and the grey area indicates the 95% confidence interval.


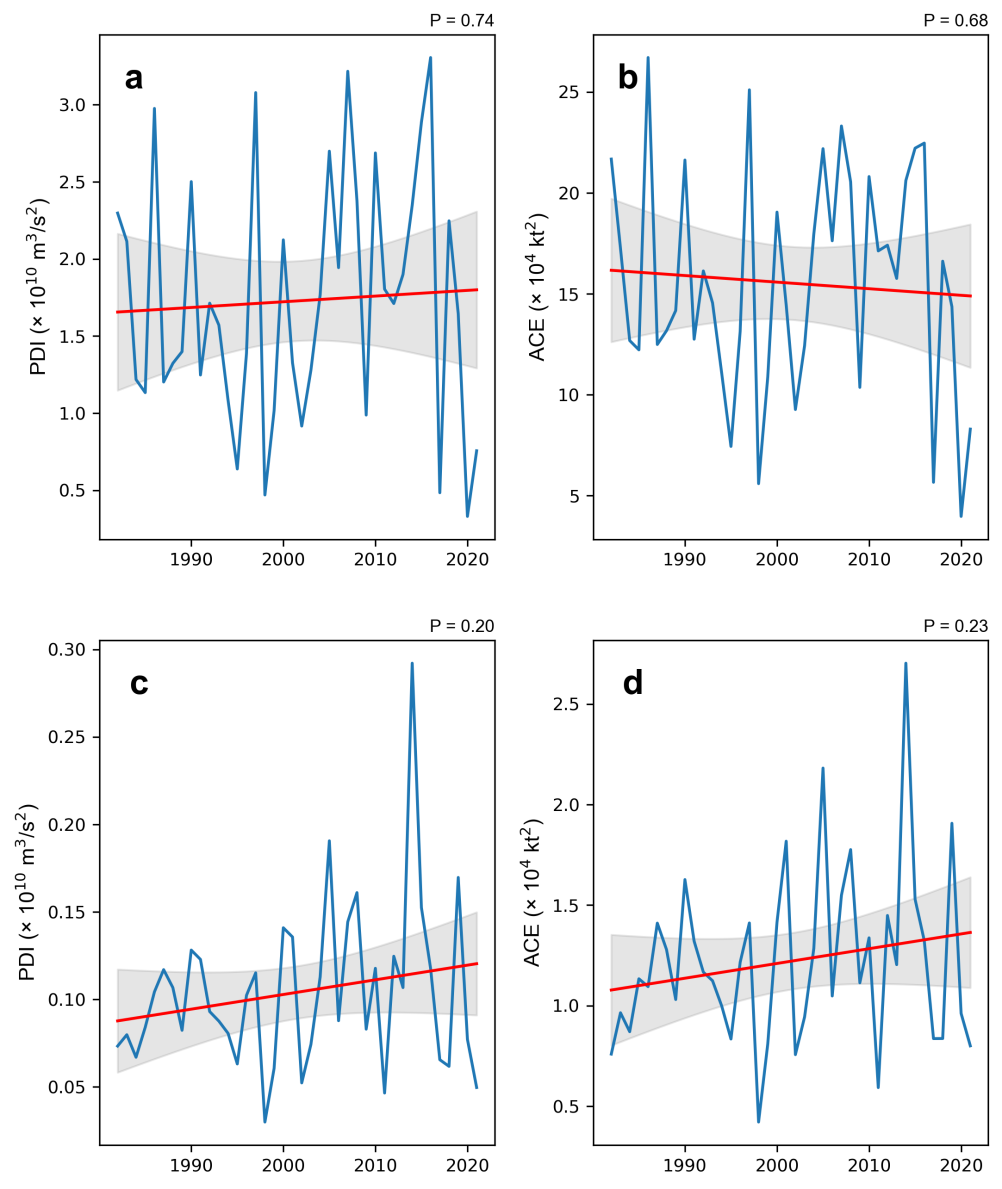


**Figure S8 | Changes in the power dissipation index (PDI) and accumulated cyclone energy (ACE) of landfalling TCs in China.** (a) Annual average PDI during the entire TC lifetime. (b) Same as (a) but for the ACE. (c) Annual average PDI over land. (d) Annual average ACE over land. The red line represents the linear trend, and the grey area indicates the 95% confidence interval.


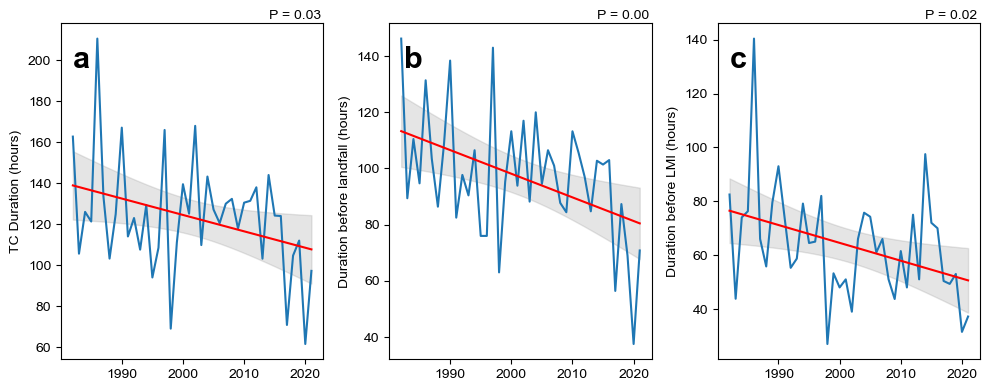


**Figure S9 | Evolution of landfalling TC duration in China during various development stages, excluding short-lived TCs (<2 days).** (a) Duration of the whole lifespan, (b) duration before landfall, (c) duration before the lifetime maximum intensity (LMI). The red line represents the linear trend, and the grey area indicates the 95% confidence interval.


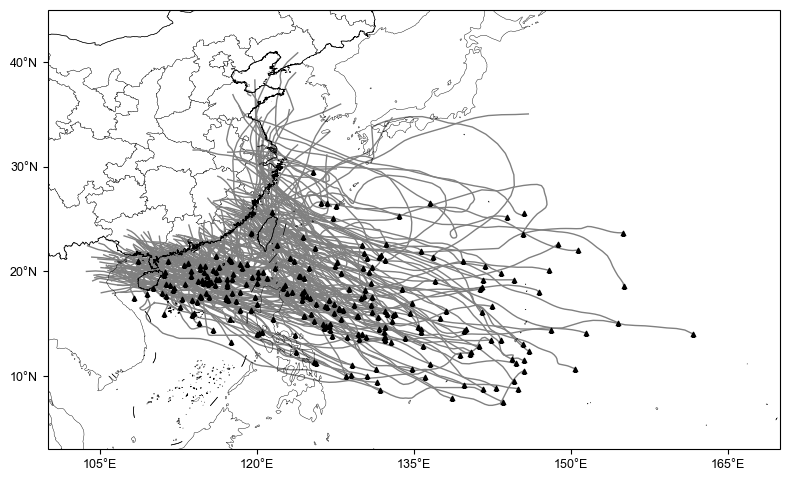


**Figure S10 | Distribution of TC tracks used in this study.** Black dots represent the genesis locations; grey curves are TC trajectories. The administrative boundaries (national and provincial) were obtained from CN Open Data (https://www.cnopendata.com/) and visualized using Cartopy and Matplotlib in Python.
